# What makes us human? A biased view from the perspective of comparative embryology and mouse genetics

**DOI:** 10.1186/1747-5333-1-16

**Published:** 2006-11-29

**Authors:** André M Goffinet

**Affiliations:** 1 Developmental Neurobiology Unit, University of Louvain Medical School, 73, Avenue Mounier, Box 7382, B1200 Brussels, Belgium

## Abstract

For a neurobiologist, the core of human nature is the human cerebral cortex, especially the prefrontal areas, and the question "what makes us human?" translates into studies of the development and evolution of the human cerebral cortex, a clear oversimplification. In this comment, after pointing out this oversimplification, I would like to show that it is impossible to understand our cerebral cortex if we focus too narrowly on it. Like other organs, our cortex evolved from that in stem amniotes, and it still bears marks of that ancestry. More comparative studies of brain development are clearly needed if we want to understand our brain in its historical context. Similarly, comparative genomics is a superb tool to help us understand evolution, but again, studies should not be limited to mammals or to comparisons between human and chimpanzee, and more resources should be invested in investigation of many vertebrate phyla. Finally, the most widely used rodent models for studies of cortical development are of obvious interest but they cannot be considered models of a "stem cortex" from which the human type evolved. It remains of paramount importance to study cortical development directly in other species, particularly in primate models, and, whenever ethically justifiable, in human.

## Report

### What makes us human?

A reader: "Gosh, who does he thinks he is, he who claims to answer that question?"

Indeed, so complex is the question that one may legitimately wonder whether it is worth asking it. A check on the internet as of this year (2006) returns different websites, from the reputed Smithsonian Institution to some that sound rather like crackpots. Most religions will tell us that Man was created by God and that our human condition will forever remain a mystery. Starting from a different viewpoint, information theory – and common sense – teach us that understanding something requires more analytical power than the object under investigation itself, thus leading to a similar conclusion. As a scientist, however, I am drawn almost inexorably to think about our "humanness" as a scientific question: even though there is no global answer, it is a question about which we can at least formulate ideas and hypotheses that can be checked by observation (e.g. fossil record) or experiment. This short commentary is aimed to those who share this endeavour.

Let me begin with two preambles. First, even the standard response "what makes us human is our brain" has obvious limitations. Imagine a creature with a human brain in the body of an ape: would she/he feel human? Would we regard her/him as human? Probably not: Being human is indeed a status that relies on, and integrates many parameters: our brain and body, of course, but also our history as a person and as a group, up to the social setting in which we live [1]. Arguably, such remarks apply to the definition of all biological species, but they are particularly striking for ours: it is not anthropomorphic to say that we have reached a unique point of sophistication in our relationships to each other and to other creatures. This remark made, I shall leave socio-biology aside – after all it is not my field – and concentrate on the point of view of brain development and evolution, about which I feel a bit less ignorant.

The other preliminary point that I wish to discuss briefly is the assumption "What makes our brain human, is our cerebral cortex, and particularly prefrontal lobes". Although I basically agree with this assertion as a first approximation, it should be noted that the evolution and development of cerebral cortical performances did not occur in isolation. Rather, the process was contingent upon the development of the nervous system and the rest of the body as a whole. To mention the most celebrated example: It is plain obvious that language is a defining trait of our species; language requires evolutionary acquisition of specific features in the larynx, and of neurological coordination of laryngeal muscles and other structures that are not related to the cerebral cortex, yet are unique to our species. The same remark can be made about the acquisition of hand skills and there are several other examples. Organisms evolve as entities. For obvious operational reasons, we mostly study the evolution of parts. But we should keep constantly in mind that, when it comes to interpretation of data, it makes little sense to discuss parts without considering the whole.

The two nuances above being made, I think most of us will agree that one of the main differences between us and other animals lays in our cognitive abilities. Every dog owner knows that animals have emotions or something close to it. They can be sad or joyous, they have their temper. They remember – sometimes at least – what they are taught. Chimpanzees can be trained, with much patience and care, to read and learn elementary symbols. There are countless anecdotes and observations indicating that elephants look depressed when loosing a mate, and may show some signs of mourning, such as returning to visit the site of death. Clearly, memory, intelligence, emotions, consciousness or even a moral sense of right and wrong are not absolutely unique to humans [2]. A recent report even claims that mice show "empathy"[3]. But only in our species have cognitive abilities reached a unique level of sophistication. As far as we know, even when compared to apes, we are the only creature who invented language, his own written rules, who runs his social life with a moral code of right and wrong, allowing us to propose that there have been some (several?) quantum leap(s) during the evolution of our brain, although there is much uncertainty about the stages in our evolution when these leaps occurred.

If we differ from animals in general, and from apes in particular, by our cognitive capacity, in which our cerebral cortex plays a key part, I would reformulate the question "What makes us human?", and ask: "What is so special about our brain and cerebral cortex"?

Man has a large brain. Some animals have larger ones, but, mostly, man has the largest brain weight when allometric correction is made for body size. Animals with the largest brains include cetaceans and elephants that we regard generally as intelligent. Interestingly, chimpanzee and gorilla score average for their brain size relative to body weight [4].

Studies of cranial endocasts indicate quite clearly that Neanderthal had larger cranial capacity and presumably larger brains than us. Brain size, although of obvious importance, is not everything. Cetaceans, even small ones such as Dolphins, have huge brains, with large and foliated cortical surfaces. Their temporal lobes are larger than ours, and this could be related to their fantastic spatial memory. Yet their cognitive skills, although far from negligible, cannot be compared to ours. The neocortex in cetaceans retains many features of its ancestral character such as a relatively low number of granular interneurons, and a relatively simple neuronal differentiation (dendrite trees). Phylogenetic isolation may have resulted in development of the nervous system chiefly by increase in nerve cell numbers (associated with great cortical expansion), by quantitative expansion without substantial architectonic evolution [5]. Neuronal numbers may not systematically vary linearly with cortical size or surface. In contrast to cetaceans, human neurons are characterized by a most elaborate architectonic organization, by a high proportion of some neuronal types, such as Cajal's "double bouquet" cell [6], and by its exquisite connectivity. Again, such parameters do not necessarily correlate with neuronal numbers and are obviously inaccessible to measurements of endocasts.

**Figure 1:**
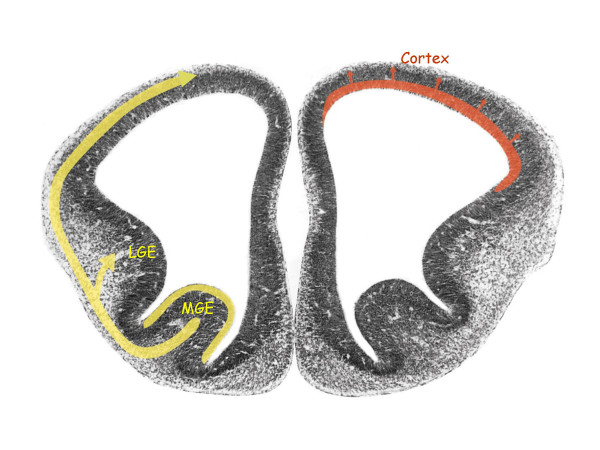
Coronal section in the forebrain of an embryonic mouse at 12.5 days of gestation (preplate stage), showing the lateral and medial ganglionic eminences (LGE, MGE) from which GABAergic interneurons migrate to the cortical anlage (left, yellow). Glutamatergic neurons destined for the cortex are generated locally in the cortical ventricular zone and migrate radially (right, red). Courtesy of V. Pachnis.

Genes that control development are preferential targets of the evolutionary process, and I would argue that the identification and study of mechanisms that regulate cortical development in different species are central to understanding our cortex. For example, developmental studies over the last ten years or so have clearly shown that the two main neuronal populations of the mammalian cortex, namely excitatory glutamatergic pyramidal cells and GABAergic interneurons are generated in different sectors of ventricular zones and migrate to the cortex along different routes. As schematized in Fig 1, the main body of cortical neurons form glutamatergic cells that migrate radially, whereas GABAergic cells generated in the ganglionic eminence, primarily the medial part (MGE), migrate to the cortex tangentially [7-9]. Whereas this developmental pattern is well established in rodents, it is also described in chick [10] and it may thus be a general feature of all amniotes. On the other hand, in the human brain, in addition to the MGE, GABAergic interneurons are also generated in the cortical VZ and migrate radially to the cortex [11]. Clearly, even though this has already been extensively studied, more works need to be done on the origin of cortical interneurons in different species.

One would think that solid data are available in the literature and can help us answer most of the comparative questions stated above? Not at all: our knowledge of comparative brain anatomy – not to mention comparative brain development – remains rudimentary [9]. This field has been neglected by grant agencies – and by most scientists – for several decades, being pursued only by a few dedicated colleagues. Yet, if we accept that the question of human origins and humanness are relevant, then I would argue that systematic comparative studies of brain anatomy and development, using state of the art techniques, are urgently needed and should be appropriately supported. A few years ago, a Human Brain Project was proposed, but I no not believe we should focus too narrowly on the human, and even on the mammalian cortex, which will be best understood in its evolutionary context. "Nothing in biology makes sense except in the light of evolution" (Dobzansky, [12]).

It is our brain that makes us human, and our brain develops under control of our genetic makeup. Hence the saying: "Our "humanness" is in our genes". This leads us to believe that, by comparing DNA sequences, we might be able to pick up human specific features and explain the whole thing. There is currently a lot of publicity about the comparison between the human and chimpanzee genomes. It is widely thought that the subtle genetic differences thus defined will point to key genetic determinants of the human species. Although these studies are fascinating and necessary, and will undoubtedly yield considerable insight, I believe this reasoning is somewhat simplistic. There is rarely an evident correlation between a genetic difference and the resulting phenotypic effects: a change that would be considered almost irrelevant may very well be most important, and vice versa. The genetic program (mostly DNA sequences) is not the phenotype, as the latter results from running the genetic program during development, and is the product of epigenetic history. During development, the epigenetic landscape unfolds in a highly non linear and massively parallel fashion, making it difficult to understand relationships a posteriori, and usually impossible to predict outcome from basic principles. Our brain evolved by natural selection, that is the survival of phenotypes (hence genomes) with the highest rate of reproduction and best suited to changing environments. The evolutionary history of the vertebrate brain is poorly understood because brain tissue does not fossilize, but also because research in comparative developmental neurobiology is still in its infancy as outlined above. The cortex may have been absent in early vertebrates: It is reduced to a periventricular layer in anamniotic vertebrates. The cortex increased in size and organization in stem amniotes, the ancestors of living reptiles, birds and mammals [9]. It gained prominence in synapsids, the lineage leading to mammals, and evolved explosively in primates. Evolution from our common ancestor with apes is only the latest major radiation in this ongoing process, and this latest step and recent brain evolution did not evolve from scratch. "Evolution is a tinkerer" [13] and can only build on a prior structure. If we want to understand brain evolution, we need to tackle DNA sequences, like the fossil record, in their historical perspective and avoid focusing narrowly. I very much doubt that we can understand the genetic control of the human brain without addressing basic genetic mechanisms in living animals that belong to as many branches of the tree as possible. It might even be possible to rescue enough DNA from fossil material, although I doubt its quality will be sufficient. Examples of what I think are elegant approaches are the work of the Haussler group on ultraconserved elements present in the genome of human and several other vertebrates [14], that led to identification of 'human accelerated regions', HAR1, and of a novel RNA gene (HAR1F) that is expressed specifically in Cajal-Retzius neurons in the developing human neocortex [15], and a recent study of human lineage-specific gene amplification [16]. But this is only an encouraging beginning and a lot remains to be done.

After the sequencing of the human and mouse genomes, obvious biomedical priorities, I thought that sequencing centers would pursue with amphioxus, a fish, a frog, a turtle, a snake or lizard, Sphenodon, chick, a crocodilian, etc... But this is not what happened ! Rather, genome sequencing is under way or almost completed for several horses, cattle, dogs, cats and other pets. As often, economic considerations and non scientific arguments prevail. But this can be changed and the price and speed of sequencing allows a wider perspective. If we believe that the questions of human origins and humanness are relevant, then concerted sequencing efforts to investigate as many branches of the tree as possible should be funded and undertaken actively. By comparing sequences on a global phylogenetic scale, we might be able to identify some of the unique, subtle genetic changes that are specific and essential to the evolution of the primate lineage and to the development of the human cortex.

The rapid increase of brain size and complexity during recent evolution in the primate lineage is well known and widely discussed elsewhere. To many, such changes in brain size in such a short time appear difficult to explain by natural selection. I believe this view is wrongly based on a sort of subconscious postulate that evolution works more or less linearly, namely that small changes in phenotypes reflect minor changes in the DNA, whereas large changes in phenotypes require huge modifications in DNA. But why should it be so, when high non linearity is in fact the rule rather than the exception. Like development, evolution works in a most non linear way. Whether this non linearity is chaotic will remain forever unknown, as we cannot rewind the tape and we have no way to produce evolution experimentally, except for a few very small and limited cases. But the point is that evolution is highly non linear, and I think some recent experimental observations can be interpreted in this frame of mind. Let me give a few examples.

The process of increased cortical surface by foliation is generally considered essential during evolution towards the human cortex. As I hinted to above, this was not the sole mechanism used by evolution to increase cortical performance, but few will doubt its importance. The process is often assumed to be complex, because it appears as such to our investigations. Yet, cortical folding can vary widely within closely related lineages, as can be appreciated by consulting the superb website "Comparative Mammalian Brain Collections" [17]. For example, in monotremes, Echidna has an elaborate, highly foliated cortex, whereas Platypus is almost lissencephalic [18]. Similar examples can be found in other phyla, including primates, some of which are almost lissencephalic. Furthermore, a mixture of brain hypertrophy with variable levels of gyrated cortex is artificially accomplished in mice by elegant, yet relatively simple manipulations [19], such as germline inactivation of caspases 3 or 9 [20,21], increased expression of beta-catenin in transgenic mice [22,23], incubation of embryonic cortex *in vitro *in the presence of lysophosphatidic acid [24,25], or manipulation of ephrin/Eph signaling [26]. Intriguingly, mice with inactivation of the phosphatase PTEN [27], and with brain-specific inactivation of alpha-catenin [28] have brain hypertrophy, but no or very little increased cortical foliation, showing that brain size, cell numbers and foliation are not always correlated. The cerebellum of Mormyrid fishes, with its large size and extensive foliation [4], provides yet another example indicating that huge increase in surface of a cortex can probably evolve or be produced quite easily.

These observations suggest that the production of a gyrated surface does not need extensive genetic changes, and could have evolved in any phylum. But it did not evolve very often, and I see at least two reasons for this, that can be tested experimentally or by comparative studies. First, acquiring a large foliated cortex may be relatively easy, but it may be more difficult to make this large cortex work efficiently. Transgenic mice with increased cortex are often not viable and, although this remains to be studied in detail, the resulting large cortex does probably not work well. This illustrates the point made above, that evolution of the brain does not occur in isolation but in the context of the whole organism. An increase in cortical size must be accompanied with balanced growth of the mesodermal components that support and vascularize the brain. Also, the increase in cortical surface and the organization of many adjacent radial cortical columns may increase neuronal excitability and susceptibility to seizures. A consequence of a highly geometrical arrangement of radial cortical columns is to facilitate modifications of the membrane potential by field effects ("ephaptic" interactions), largely believed to be involved in the oscillations of electrocortical rhythms and in generation of seizures [29]. This quasi crystalline arrangement presumably has advantages in terms of computational power, but also comes at a price, as ephaptic excitation facilitates the tangential spreading of activity and decreases the threshold for aberrant epileptic discharges. The second reason, not in contradiction with the first, may be more important. Namely, the acquisition of a large brain and particularly of a large foliated cortex may not be an evolutionary advantage *per se*. Most species that are hugely successful in the evolutionary sense do not have a large brain or high cognitive power. Large brain size and increased computational power presumably proved evolutionary useful quite recently, in early Homo, and the reason why this parameter was selected positively at some point in our phylogenetic history remains unknown. Perhaps, like several traits, increased cortical surface was just "tried" at some point in the primate lineage and, once the track had been taken, there was little choice but to keep the option, because it would have cost much more to turn back than to continue. The option finally paid off, as humans are obviously a very successful species, at least at this moment and provided we do not end it all ourselves by burning or exploding the planet.

Contrary to common belief, stem mammals probably did not have a rodent-like forebrain. Rodents are highly evolved animals that are not directly related to stem mammals, from which the lineage leading to primates is in fact more directly derived [30]. Although this is not proven, I consider it likely that stem mammals had a relatively unspecialized, basic cortex, possibly with some foliation, from which highly specialized lissencephalic cortices evolved in lineages such as rodent, whereas other lineages kept some foliation and even increased it in some branches, most notably ours. It is generally accepted that evolution works more easily on relatively undifferentiated forms [31] and that neoteny of primates was a contributing factor to their rapid evolution. If this view is correct, then the mouse cortex is not a model of the "primitive" cortex of stem mammals, and inferences from mouse data in terms of cortical evolution should be made with appropriate caution.

Another illustration that the mouse, with all its advantages, should not be considered the sole model for cortical development and evolution concerns the role of Cajal-Reztius cells, early neurons in the cortical marginal zone that degenerate massively around birth [32]. Studies in reeler and other mutant mice and human genetic studies clearly demonstrate that Reelin secreted by Cajal-Retzius cells is absolutely required for normal cortical development in mice and for foliation of the human cortex [33]. Yet, in mice, genetic ablation of most of Cajal-Retzius cells does not perturb cortical development much, indicating a large redundancy [34]. Although this remains to be studied further, it seems that there is a huge excess of Cajal-Retzius cells and of Reelin in rodents, and that this is not the case in the human, where a provision of Reelin seems to be provided over an extended period of time in the marginal zone [35].

As discussed above, the unique cognitive power of the human brain seems to be due to the evolutionary acquisition of multiple factors such as high neuronal number, large foliated cortex, optimal architectonic organization, complex neuronal types, highly organized and elaborate connections. The evolution of the human brain has proceeded at amazing speed. We have reached a stage where our cognitive capacity increases more through technological innovation, and cultural evolution vastly outpaces biological evolution. However, I see no reason why brain evolution should stop at the present human level and it is impossible to escape the question of its future. Without going into science fiction, I would like to mention one feature of the cerebral cortex that receives scant attention – it is in fact generally ignored – and that I find very intriguing, namely the subpial granular layer (SGL). The SGL, is a transient contingent of cells that are apparently generated from a basal region, close to the hilus of the sylvian fissure and the paleoventricle, and migrate tangentially in the subpial cortical marginal zone during mid- and late gestation. The SGL is much more developed in human than other mammals, to the point that it is sometimes considered human-specific, even though a diminutive SGL has now been described in other mammals. Initially described more than a century ago by Ranke, the SGL was examined in some detail by Brun [36], who concluded that its cells differentiate into glia and/or probably die after entering the cortical ribbon radially. A more recent study indicated that the SGL cells are likely neuronal and enter the cortex radially [37], but remained inconclusive about their fate. By analogy with the development of the external and internal granular layers of the cerebellum, a reasonable hypothesis could be that subpial neurons contribute to the cortical neuronal population but that this went undetected because it represents a minority of neurons. The SGL might provide a source of interneurons in addition to the main contingent that originates from the ganglionic eminences. Like in the cerebellum, an increase in SGL cells could result in an increase in surface and folding of the cerebral cortical surface and, who knows, result in a cerebral cortex with increased computational power. Even though the SGL is best studied in man, the idea that it could play a role during cortical development and evolution is not pure speculation. Some diminutive SGL is present in mammalian models and techniques are available to define better the cellular constitution of the SGL and the fate of its cells after they enter the cortex, to identify their repertoire of gene expression, the transcription factors implicated in their differentiation.

I am convinced that we should not limit ourselves to the analysis of the mouse and non mammalian species, but that we should also study actively brain development in primates, and particularly in human, using state of the art techniques. Of course, ethical considerations are of the utmost importance and must be taken into account. But studies of brain development in primates and man, using the whole arsenal of modern technology are a unique way to trace novel, original avenues and to address scientifically the question of the evolution of our cerebral cortex, and of our biological nature.

## Conclusion

The human cerebral cortex is at the core of human nature. Our cortex evolved from that in stem amniotes and cannot be understood if excluded from this evolutionary context. If we want to understand better our cerebral cortex, more efforts should be invested in comparative studies of embryonic development, using state of the art technologies. Genomic sequencing efforts should be directed at all branches of the vertebrate tree rather than focused narrowly on mammals. Finally, the rodent cortex is not a perfect model of the stem mammalian cortex and specific studies of primate and human cortices are necessary. In addition to its fundamental interest, an improved scientific knowledge of human nature will help us define better our place and thus our rights and our duty in relation to our environment and to ourselves. This is after all the ultimate ecological challenge!
